# Do PCSK9 Inhibitors Impair Memory? A Dual Approach Combining Real-World Data and Genetic Evidence

**DOI:** 10.3390/pharmacy13050125

**Published:** 2025-09-03

**Authors:** Xuezhong Shi, Shijia Wang, Yongli Yang, Xudong Xia, Jingwen Fan, Jingjing Wang, Nana Wang, Xiaocan Jia

**Affiliations:** 1 Department of Epidemiology and Biostatistics, College of Public Health, Zhengzhou University, Zhengzhou 450001, China; xzshi@zzu.edu.cn (X.S.); sjwang2000@gs.zzu.edu.cn (S.W.); ylyang377@zzu.edu.cn (Y.Y.); hmpaxxd@126.com (X.X.); fjw@gs.zzu.edu.cn (J.F.); wjj95524@163.com (J.W.); wnn0924@126.com (N.W.); 2 Center for Drug Evaluation of Henan, Zhengzhou 450002, China

**Keywords:** PCSK9 inhibitors, memory impairment, amnesia, pharmacovigilance, Mendelian randomization

## Abstract

Emerging evidence suggested a potential link between lipid-lowering therapies and neurocognitive effects, raising concerns regarding the possible adverse impact of PCSK9 inhibitors on memory loss. We extracted adverse events associated with memory loss for PCSK9 inhibitors from the Food and Drug Administration Adverse Event Reporting System (FAERS), covering the period from the first quarter (Q1) of 2022 to Q1 of 2025. Reporting odds ratio (ROR), Medicines and Healthcare Products Regulatory Agency (MHRA), empirical Bayesian geometric mean (EBGM), and information component (IC) were used for pharmacovigilance analysis. Drug target Mendelian randomization (MR) was utilized to assess the causal association between PCSK9 inhibitors and memory loss. A total of 389 occurrences of memory loss associated with PCSK9 inhibitors were recorded among 388 patients. In the pharmacovigilance analysis, memory loss did not show a significant signal for PCSK9 inhibitors in both the full dataset [ROR (95% CI): 0.79 (0.72, 0.88); PRR = 0.79, χ^2^ = 20.64; EBGM05 = 0.73; IC025 = −2.00] and the lipid-lowering targets dataset [ROR (95%CI): 0.59 (0.53, 0.66); PRR = 0.59, χ^2^ = 95.33; EBGM05 = 0.59; IC025 = −2.30]. The drug target MR revealed no causal association between PCSK9 inhibitors and memory loss (*p* < 0.05). The present study failed to establish a causal relationship between PCSK9 inhibitors and memory loss. By providing both real-world and genetic evidence, our findings might help alleviate concerns and support the notion that PCSK9 inhibitors were relatively safe regarding memory function.

## 1. Introduction

Proprotein convertase subtilisin–kexin type 9 (PCSK9) inhibitors have been demonstrated to have significant efficacy in reducing low-density lipoprotein cholesterol (LDL-C) by promoting the recycling of LDL receptors on the liver surface [1]. Clinical trials have established the potent LDL-C lowering efficacy of PCSK9 inhibitors (evolocumab, alirocumab, and inclisiran), demonstrating exceeding 50% reductions from the baseline [2,3,4]. Nevertheless, accumulating evidence has raised concerns regarding the potential neurocognitive issues associated with lipid-lowering targets [5]. The neurocognitive safety signal previously observed with statin therapy appeared potentially applicable to PCSK9 inhibitors, warranting thorough clinical assessment [6]. Notably, the U.S. Food and Drug Administration (FDA) has issued a safety warning regarding the potential adverse effects of memory loss linked to PCSK9 inhibitors, underscoring the need for further investigation into this potential effect [7].

Memory loss was a condition affecting individuals with memory difficulties [8]. The OSLER-1 trial and ODYSSEY LONG TERM trial indicated that the incidence of memory loss was higher in patients treated with PCSK9 inhibitors compared to those receiving placebo [9,10]. However, in a systematic review of randomized controlled trials (RCTs), Akshay Machanahalli Balakrishna et al. [11] found that PCSK9 inhibitors therapy, which lowered LDL-cholesterol to ultra-low levels (<40 mg/dL), was not associated with an increased risk of neurocognitive adverse events (AEs) compared with control groups maintaining LDL-C ≥ 40 mg/dL. Critically, it must be recognized that inherent limitations of clinical trials, such as reporting and selection bias, restricted sample sizes, and short follow-up durations, rendered these studies underpowered for comprehensive, long-term safety assessment in large-scale real-world populations [12]. Investigating the relationship between PCSK9 inhibitors and memory loss in general real-world populations, coupled with causal inference analysis, constitutes an urgent research need.

Pharmacovigilance analysis leveraged the FDA Adverse Event Reporting System (FAERS), which covered a wide range of populations and facilitated long-term monitoring of AEs to identify potential safety concerns [13]. Drug target Mendelian randomization (MR) used genetic variants near drug target genes as proxies to mimic the effects of pharmacological intervention [14]. It enabled causal inference with enhanced robustness against confounding and reverse causality. The integration of pharmacovigilance analysis with drug target MR could establish a robust causal inference framework [15].

This study aimed to explore the potential association between PCSK9 inhibitors and memory loss by employing pharmacovigilance analysis and drug target MR. Our research holds important clinical implications for the administration of PCSK9 inhibitors and the monitoring of side effects in future PCSK9 inhibitors’ therapies.

## 2. Materials and Methods

### 2.1. Study Overview

**Figure 1:**
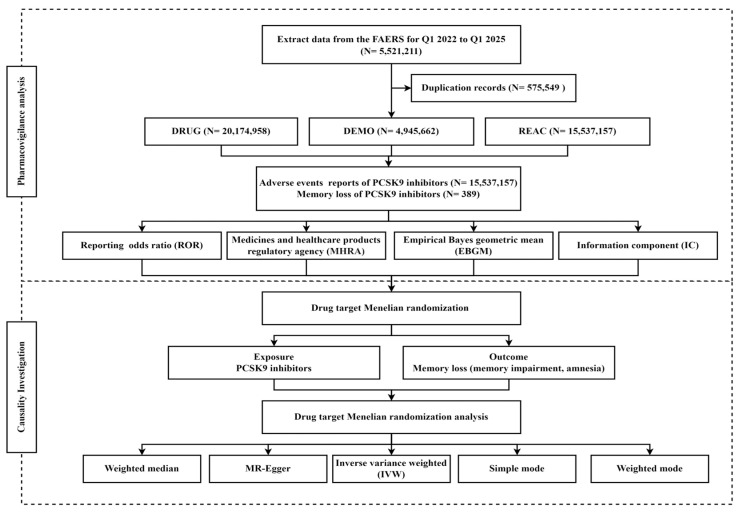
Flowchart of the study.

In this study, pharmacovigilance analysis and drug target MR analysis were combined (Figure 1). Pharmacovigilance analysis was pulled from the FAERS to examine the association between PCSK9 inhibitors and memory loss. Based on the pharmacovigilance findings, MR analysis was employed to verify the causal relationship between the PCSK9 inhibitors and memory loss.

### 2.2. Pharmacovigilance Analysis

From the first quarter (Q1) 2022 to Q1 2025, a total of 4,945,662 unique cases and 15,537,157 AEs were extracted from the FAERS for a retrospective pharmacovigilance disproportionality analysis. PRIMARYID, CASEID, and FDA_DT fields from the DEMO table were retrieved and sorted by CASEID, FDA_DT, and PRIMARYID. In instances where CASEID was identical, the latest FDA_DT was chosen. When both FDA_DT and CASEID were the same, the highest PRIMARYID was retained. AEs related to memory loss were classified and described according to high-level terms (HLT) and preferred terms (PT) in the 27th version of the Medical Dictionary for Regulatory Activities (MedDRA). The search terms for target drugs contained their brand or generic names: ‘inclisiran’, ‘alirocumab’, ‘evolocumab’, ‘pcsk9’, ‘praluent’, ‘leqvio’, ‘repatha’. Memory loss encompassed memory impairment, amnesia, amnestic disorders, anterograde amnesia, Korsakoff’s syndrome, post-traumatic memory disorder, retrograde amnesia, transient global amnesia, and Wernicke–Korsakoff syndrome as classified by MedDRA.

Based on the 2 × 2 table of the proportional imbalance algorithm (Table S1), reporting odds ratio (ROR), Medicines and Healthcare Products Regulatory Agency (MHRA), empirical Bayesian geometric mean (EBGM), and information component (IC) were employed to evaluate the association between PCSK9 inhibitors and memory loss. A significant signal for the ROR was identified when the lower limit of the 95% confidence interval (CI) > 1 and there were at least 3 reported AEs [16]. A significant signal under the MHRA framework required a proportional reporting ratio (PRR) ≥ 2, a chi-square (*χ*^2^) ≥ 4 [17]. Statistical significance was defined by the lower limit of the 95% CI of the EBGM (EBGM05) ≥ 2 and the lower limit of the 95% CI of the IC (IC025) > 0 [18]. The consistent signal detection across four disproportionality methods would indicate memory loss as a potential positive signal for PCSK9 inhibitors.

To detect the signal of PCSK9 inhibitors more accurately, this study designed two signal detection backgrounds: the full dataset background and the lipid-lowering drugs background by the Anatomical Therapeutic Chemical (ATC) classification system. Additionally, stratification analyses were conducted based on gender and age to enhance the precision of signal evaluation. The effects of each PCSK9 inhibitor on memory were also examined. Serious outcomes were characterized as including death, hospitalization, life-threatening, disability, and other serious outcomes. All the pharmacovigilance disproportional analyses were conducted in the SAS 9.4 software.

### 2.3. MR Analysis

Given the clear clinical efficacy of PCSK9 inhibitors in reducing LDL-C levels, single nucleotide polymorphisms (SNPs) associated with LDL-C were utilized as instrumental variables (IVs) sourced from the Global Lipids Genetics Consortium (GLGC) [19]. The genomic region chr1: 55505221–55530525, which encompassed genes and regulatory elements associated with PCSK9 inhibitors, was selected for the identification of SNPs that serve as proxies for PCSK9 inhibitors. The sequence information about this region was sourced from the National Center for Biotechnology Information (NCBI) database. SNPs strongly associated with LDL-C levels (*p* < 5 × 10^−8^) and exhibiting lower linkage disequilibrium (LD) were selected with a clumping r^2^ cutoff of 0.3 and a clumping distance of 100 kilobases. Finally, 88 SNPs were retained.

GWAS data for memory loss, memory impairment, and amnesia were sourced from the FinnGen R12 database, the Veterans Affairs Million Veteran Program, and the UK Biobank, respectively. Detailed information regarding the GWAS data for each outcome is presented in Table S2. As expected, in positive control analyses, genetically proxied PCSK9 inhibitors reduced the risk of coronary heart disease (CHD). GWAS data for CHD were extracted from CARDIoGRAMplusC4D, which primarily consisted of individuals of European ancestry [20].

In drug target-based MR analysis, inverse-variance weighted (IVW) analysis was employed as the primary approach. When all IVs were valid, an unbiased estimation of causality was yielded [21]. As the IVW approach was susceptible to outliers and horizontal pleiotropy, four complementary analyses including MR-Egger regression, weighted median, simple mode, and weighted mode were conducted to generate unbiased estimates. The MR-Egger method could provide a consistent estimation of the causal effect, under the weaker assumption of the InSIDE (INstrument Strength Independent of Direct Effect) assumption [22]. When at least 50% valid IVs were valid, the weighted median method provided a reasonable estimation. In addition, the simple mode and weighted mode methods could be utilized as supplementary sensitivity analyses. These approaches have been demonstrated to enhance robustness against directional pleiotropy in cases where the InSIDE assumption was violated or when the proportion of valid instrumental variables (IVs) remained uncertain [23]. All the MR statistical analyses were used in the R 4.4.1 software.

## 3. Results

### 3.1. Descriptive Analysis of the FAERS

A total of 388 patients reported experiencing memory loss associated with the use of PCSK9 inhibitors (Table 1). The proportion of evolocumab-related memory loss (67.01%) was higher than alirocumab (13.14%) and inclisiran (19.85%). The proportion of female patients (68.30%) exceeded that of male patients (27.06%). The majority of patients were aged 65 years or older (51.03%), reported by consumers (82.22%), and sourced from the United States (89.95%).

### 3.2. Pharmacovigilance Analysis of the FAERS

A total of 389 cases of memory loss associated with PCSK9 inhibitors have been reported, with memory impairment and amnesia being the most frequently documented adverse effects in the FAERS. Table 2 and Table 3 showed disproportionality analysis results for PCSK9 inhibitor-associated memory loss, meeting the minimum case threshold (≥3 reports), based on the full dataset and lipid-lowering drugs dataset, respectively. Across four disproportionality analysis methods, no significant association was observed between PCSK9 inhibitors and memory loss both the full dataset [ROR (95%CI): 0.79 (0.72, 0.88); PRR = 0.79, χ^2^ = 20.64; EBGM05 = 0.73; IC025 = −2.00] and the lipid-lowering targets dataset [ROR (95%CI): 0.59 (0.53, 0.66); PRR = 0.59, χ^2^ = 95.33; EBGM05 = 0.59; IC025 = −2.30]. No significant association was found between each PCSK9 inhibitor and memory loss (Tables S3 and S4). Stratification analyses by gender and age yielded results consistent with the main analysis (Tables S5 and S6).

### 3.3. MR Analysis for PCSK9 Inhibitors on Memory Loss

Genetically predicted PCSK9 inhibitors were significantly associated with a lower risk of CHD, using the IVW method [OR (95%CI): 0.52 (0.47, 0.58)] (Table 4). The positive control analysis was performed to validate the effectiveness of our instruments. The IVW result did not find the association between PCSK9 inhibitors and memory loss (*p* > 0.05) (Table 5).

## 4. Discussion

Both pharmacovigilance analysis and drug target MR analysis found no evidence supporting a causal relationship between PCSK9 inhibitors and memory loss. These consistent findings across real-world observations and genetic investigations might provide reassurance to clinicians regarding the safety of PCSK9 inhibitors regarding memory function.

Real-world pharmacovigilance analysis has mitigated clinical trial limitations through ongoing safety monitoring and facilitated the identification of delayed or low-incidence safety signals across diverse populations. Previous pharmacovigilance analysis of psychiatric AEs linked to PCSK9 inhibitors (alirocumab and evolocumab), using FAERS data from the third quarter (Q3) 2015 to Q1 2023 and applying both MHRA and IC methods, identified memory loss as a highly significant signal [24]. Nevertheless, other pharmacovigilance studies analyzing the FAERS data using both the ROR and the MHRA methods from the Q3 of 2015 to the second quarter (Q2) of 2021 did not identify memory loss as a significant positive signal for PCSK9 inhibitors (alirocumab and evolocumab) [25]. A similar finding was also reported by Chunmei Ji et al. [26], who utilized the ROR method from the FAERS database, covering the period from Q3 of 2015 to Q1 2021. This discrepancy might be attributed to the different signal mining methods and the differing coverage periods of the FAERS data. In addition, previous studies on PCSK9 inhibitors were limited to alirocumab and evolocumab, without consideration of inclisiran. To address these limitations, our study employed four quantitative signal detection methods (ROR, MHRA, EBGM, and IC) and extracted the most recent FAERS data to investigate the association between all available PCSK9 inhibitors (alirocumab, evolocumab, and inclisiran) and memory loss. Our pharmacovigilance analysis revealed no significant association between PCSK9 inhibitors and memory loss.

Through the innovative methodology of drug target MR, we simulated the pharmacological effects of PCSK9 inhibitors while effectively controlling for confounding factors [27]. This approach enabled causal inference regarding genetic susceptibility to memory loss associated with PCSK9 inhibitors. Andrew S Bell and colleagues [28], employing the drug target MR, demonstrated that genetically proxied PCSK9 inhibitors exhibited a neutral profile with respect to neuropsychiatric side effects. Building upon these findings, our drug target MR study specifically evaluated memory-related outcomes and found no causal relationship between PCSK9 inhibitors and memory loss, which was consistent with our pharmacovigilance analysis. Furthermore, a comparative analysis conducted by Rosoff et al. [29] demonstrated that while PCSK9 inhibitors showed no significant association with memory performance, HMG-CoA reductase inhibitors (commonly known as statins) exhibited statistically significant adverse neurocognitive effects. These results suggested that memory loss may be more strongly associated with HMG-CoA reductase inhibitors than with PCSK9 inhibitors, though further studies are needed to confirm this relationship.

Our results indicated that among patients experiencing memory-related AEs associated with PCSK9 inhibitors, a higher proportion were female and elderly individuals (≥65 years old). However, the stratification analyses did not reveal a statistically significant association between PCSK9 inhibitors and memory loss across both age and gender. The stratification results were in accordance with a hospital registry study that stratified by age and sex, which did not reveal any association between PCSK9 inhibitors and neuropsychiatric events [30]. Despite the lack of a clear pharmacological signal, women and the elderly exhibited well-documented physiological characteristics, including their tolerability to opioid deprescribing as well as age-related alterations in the blood–brain barrier [31,32]. These factors may enhance drug exposure and susceptibility within the central nervous system. Given the physiological particularities and potential vulnerabilities in female and elderly individuals, a cautious and individualized approach to treatment is warranted in practice.

There were several reasons to suggest alleviating the worries and clarifying that PCSK9 inhibitors were unlikely to result in memory loss. PCSK9 inhibitors exerted their effects extracellularly by targeting the PCSK9 protein in the bloodstream, thereby preventing LDL receptor degradation primarily in the liver [33]. In contrast, statins inhibited cholesterol synthesis throughout the body, including potentially within the central nervous system (CNS), where cholesterol was crucial for the formation of neuromyelin sheaths, neurological signaling, mitochondrial function, and synaptic development [34]. Furthermore, unlike statins, which were small molecules capable of penetrating the blood–brain barrier (BBB), PCSK9 inhibitors were too large to efficiently cross the BBB, thus limiting direct brain effects [35]. Therefore, although the FDA initially raised concerns regarding possible neurocognitive AEs caused by PCSK9 inhibitors, accumulated evidence indicated that the likelihood of these drugs causing severe memory loss is low. However, this could not completely rule out the possibility of minor effects.

In the real world, the effects of intensive lipid-lowering therapies, particularly those aimed at reducing cholesterol levels, on neurocognitive function require careful and multi-level consideration. This is particularly important in relation to alterations in cell membrane lipid composition, synaptic integrity, and the efficiency of β-amyloid clearance [36]. Large-scale, long-term studies are needed to assess the risks of intensive lipid-lowering therapies on memory loss. Controlled studies comparing the short- and long-term effects of various lipid-lowering medications on cognitive function are also warranted.

Unlike conventional pharmacovigilance approaches that rely solely on spontaneous reporting systems, our study incorporated drug target MR analysis, thereby enhancing the ability to differentiate true drug effects from confounding factors. Furthermore, in contrast to previous studies that predominantly concentrated on older PCSK9 inhibitors such as evolocumab and alirocumab, our investigation included the newer siRNA agent inclisiran, providing a more comprehensive overview of the current PCSK9 therapeutic landscape. Agent-specific analyses for each PCSK9 inhibitor and extensive stratification analyses across age and gender yielded consistent results, reinforcing the robustness and generalizability of our findings.

Nevertheless, some limitations of this research should be noted. The monitoring of memory-related AEs in this study relied on the clinical trial’s spontaneous reporting system, a standard for pharmacovigilance that captures patient-reported experiences. However, it is important to note that for complex endpoints like neurocognitive function, spontaneous reporting may lack sensitivity and underestimate the true incidence of these events. Spontaneous reporting of memory-related AEs may lead to underreporting and misclassification, potentially biasing results. Therefore, we further adopted drug target MR analysis to reduce the common confounding bias and reverse causality problems in traditional observational studies, providing more robust evidence to support causal inference. In addition, the current investigation primarily utilized GWAS data derived from individuals of European ancestry and FAERS data representing a mixed ancestry of European and Latin ancestry. The differences in the racial composition of the populations in these two databases might potentially affect the consistency of the results. In future research, it is essential to give priority to exploring potential variations among different racial groups.

## 5. Conclusions

Synthesizing pharmacovigilance analysis and drug target validation, this study indicated a lack of evidence for a correlation between PCSK9 inhibitors and memory loss. Convergent real-world and genetic evidence substantiated the memory safety of these agents, potentially mitigating prior FDA warning concerns. Longitudinal surveillance in vulnerable populations is still advised to exclude rare or long-term risks.

## Figures and Tables

**Table 1:** Characteristics of patients treated with PCSK9 inhibitors experiencing memory loss.

Characteristics	PCSK9 Inhibitors (*n* = 388)
Memory Loss, [*N* = 389(%)]	
Memory impairment	307 (78.92%)
Amnesia	79 (20.31%)
Transient global amnesia	2 (0.51%)
Amnestic disorder	1(0.26%)
PCSK9 inhibitors, [*n* (%)]	
Alirocumab	51 (13.14%)
Evolocumab	260 (67.01%)
Inclisiran	77 (19.85%)
Gender, [*n* (%)]	
Male	105 (27.06%)
Female	265 (68.30%)
Missing	18 (4.64%)
Age (years), [*n* (%)]	
18–64	51 (13.14%)
≥65	198 (51.03%)
Missing	139 (35.83%)
Median (IQR)	72.00 (67.75–77.00)
Reported person, [*n* (%)]	
Consumer	319 (82.22%)
Health professional	42 (10.82%)
Physician	20 (5.15%)
Pharmacist	7 (1.81%)
Reported countries, [*n* (%)]	
United States	349 (89.95%)
Other country	39 (10.05%)
Reported year, [*n* (%)]	
2022	26 (6.70%)
2023	29 (7.47%)
2024	40 (10.31%)
2025 Q1	10 (25.77%)
Serious outcomes, [*n* (%)]	
Hospitalization	51 (28.81%)
Disability	10 (5.65%)
Life-threatening	3 (1.69%)
Other serious outcomes	113 (63.84%)

Abbreviations: *N*, number of adverse events; *n*, number of cases.

**Table 2:** The signal strength of memory loss for PCSK9 inhibitors in the full dataset.

AEs	*N*	ROR (95% CI)	MHRA		EBGM05	IC025
			PRR	χ^2^		
Memory loss	389	0.79 (0.72, 0.88)	0.79	20.64	0.73	−2.00
Memory impairment	307	0.85 (0.76, 0.96)	0.85	7.63	0.78	−1.89
Amnesia	79	0.63 (0.51, 0.79)	0.63	16.97	0.53	−2.33

Abbreviations: AEs, adverse events; CI, confidence interval; ROR, reporting odds ratio; MHRA, Medicines and Healthcare Products Regulatory Agency; PRR, proportional reporting ratio; EBGM05, lower limit of the 95% CI of the empirical Bayesian geometric mean; IC025, lower limit of the 95% CI of the information component.

**Table 3:** The signal strength of memory loss for PCSK9 inhibitors in the lipid-lowering drugs dataset.

AEs	*N*	ROR (95% CI)	MHRA		EBGM05	IC025
			PRR	χ^2^		
Memory loss	389	0.59 (0.53, 0.66)	0.59	95.33	0.59	−2.30
Memory impairment	307	0.72 (0.64, 0.82)	0.72	27.45	0.69	−2.05
Amnesia	79	0.35 (0.28, 0.44)	0.35	88.99	0.33	−3.00

Abbreviations: AEs, adverse events; CI, confidence interval; ROR, reporting odds ratio; MHRA, Medicines and Healthcare Products Regulatory Agency; PRR, proportional reporting ratio; EBGM05, lower limit of the 95% CI of the empirical Bayesian geometric mean; IC025, lower limit of the 95% CI of the information component.

**Table 4:** MR analysis for PCSK9 inhibitors with CHD.

Exposure	Outcome	MR Methods	SNPs	OR (95% CI)	*p*
PCSK9 inhibitors	CHD	IVW	56	0.52 (0.47, 0.58)	<0.001
		MR-Egger	56	0.52 (0.43, 0.63)	<0.001
		Weighted median	56	0.55 (0.47, 0.65)	<0.001
		Simple mode	56	0.43 (0.33, 0.57)	<0.001
		Weighted mode	56	0.56 (0.47, 0.67)	<0.001

Abbreviations: CHD, coronary heart disease; IVW, Inverse variance weighted; CI, confidence interval.

**Table 5:** MR analysis for PCSK9 inhibitors with memory loss.

Exposure	Outcome	MR Methods	SNPs	OR (95% CI)	*p*
PCSK9 inhibitors	Memory loss	IVW	81	0.98 (0.78, 1.23)	0.837
		MR-Egger	81	0.94 (0.69, 1.29)	0.718
		Weighted median	81	0.95 (0.71, 1.27)	0.734
		Simple mode	81	1.30 (0.68, 2.49)	0.430
		Weighted mode	81	1.01 (0.79, 1.29)	0.953
	Memory impairment	IVW	83	1.20 (0.96, 1.49)	0.116
		MR-Egger	83	0.89 (0.64, 1.25)	0.514
		Weighted median	83	1.05 (0.77, 1.43)	0.748
		Simple mode	83	1.28 (0.69, 2.37)	0.435
		Weighted mode	83	1.11 (0.82, 1.50)	0.488
	Amnesia	IVW	75	1.75 (0.74, 4.14)	0.204
		MR-Egger	75	3.07 (0.85, 11.12)	0.092
		Weighted median	75	2.51 (0.61, 10.35)	0.203
		Simple mode	75	2.17 (0.18, 26.97)	0.548
		Weighted mode	75	2.17 (0.65, 7.28)	0.212

Abbreviations: IVW, Inverse variance weighted; CI, confidence interval.

## Data Availability

The pharmacovigilance data were openly available from the FDA Adverse Event Reporting System (https://www.fda.gov/drugs/drug-approvals-and-databases/fda-adverse-event-reporting-system-faers-database, accessed on 15 May 2025). The drug target MR data were from the Global Lipids Genetics Consortium (https://csg.sph.umich.edu/willer/public/glgc-lipids2021, accessed on 16 May 2025) and FinnGen R12 database (https://www.finngen.fi/en, accessed on 16 May 2025).

## Supplementary Materials

The following supporting information can be downloaded at: https://www.mdpi.com/article/10.3390/pharmacy13050125/s1, Table S1: 2 × 2 table of disproportionality analysis; Table S2: The overview of the GWAS data; Table S3: The signal strength of memory loss for each PCSK9 inhibitor in the full dataset; Table S4: The signal strength of memory loss for each PCSK9 inhibitor in the lipid-lowering drugs dataset; Table S5: The stratification analysis of PCSK9 inhibitors in the full dataset; Table S6: The stratification analysis of PCSK9 inhibitors in the lipid-lowering drugs dataset.

## Abbreviations

The following abbreviations are used in this manuscript:

PCSK9	Proprotein convertase subtilisin–kexin type 9
AEs	Adverse events
LDL-C	Low-density lipoprotein cholesterol
FAERS	U.S. Food and Drug Administration Adverse Event Reporting System
MR	Mendelian randomization
RCTs	randomized controlled trials
ROR	Reporting odds ratio
PRR	Proportional reporting ratio
IC	Information component
IC025	Lower limit of the 95% CI of the IC
MHRA	Medicines and Healthcare Products Regulatory Agency
EBGM	Empirical Bayesian geometric mean
EBGM05	Lower limit of the 95% CI of the EBGM
MedDRA	Medical Dictionary for Regulatory Activities
ATC	Anatomical Therapeutic Chemical
NCBI	National Center for Biotechnology Information
SNPs	Single nucleotide polymorphisms
IVs	Instrumental variables
GLGC	Global Lipids Genetics Consortium
CHD	Coronary heart disease
LD	Linkage disequilibrium
IVW	Inverse-variance weighted
HLT	High-level terms
PT	Preferred terms
Q1	First quarter
Q2	Second quarter
Q3	Third quarter
CNS	central nervous system
BBB	Blood–brain barrier

## References

[1] Barale C., Melchionda E., Morotti A., Russo I. (2021). PCSK9 Biology and Its Role in Atherothrombosis. Int. J. Mol. Sci..

[2] Sabatine M.S., Giugliano R.P., Keech A.C., Honarpour N., Wiviott S.D., Murphy S.A., Kuder J.F., Wang H., Liu T., Wasserman S.M. (2017). Evolocumab and Clinical Outcomes in Patients with Cardiovascular Disease. N. Engl. J. Med..

[3] Schwartz G.G., Steg P.G., Szarek M., Bhatt D.L., Bittner V.A., Diaz R., Edelberg J.M., Goodman S.G., Hanotin C., Harrington R.A. (2018). Alirocumab and Cardiovascular Outcomes after Acute Coronary Syndrome. N. Engl. J. Med..

[4] Raal F.J., Kallend D., Ray K.K., Turner T., Koenig W., Wright R.S., Wijngaard P.L.J., Curcio D., Jaros M.J., Leiter L.A. (2020). Inclisiran for the Treatment of Heterozygous Familial Hypercholesterolemia. N. Engl. J. Med..

[5] Rojas-Fernandez C.H., Goldstein L.B., Levey A.I., Taylor B.A., Bittner V. (2014). An assessment by the Statin Cognitive Safety Task Force: 2014 update. J. Clin. Lipidol..

[6] Strom B.L., Schinnar R., Karlawish J., Hennessy S., Teal V., Bilker W.B. (2015). Statin Therapy and Risk of Acute Memory Impairment. JAMA Intern. Med..

[7] Banach M., Rizzo M., Nikolic D., Howard G., Howard V., Mikhailidis D. (2017). Intensive LDL-cholesterol lowering therapy and neurocognitive function. Pharmacol. Ther..

[8] Borelli C.M., Grennan D., Muth C.C. (2020). Causes of Memory Loss in Elderly Persons. JAMA.

[9] Koren M.J., Sabatine M.S., Giugliano R.P., Langslet G., Wiviott S.D., Ruzza A., Ma Y.H., Hamer A.W., Wasserman S.M., Raal F.J. (2019). Long-Term Efficacy and Safety of Evolocumab in Patients With Hypercholesterolemia. J. Am. Coll. Cardiol..

[10] McCullough P.A., Ballantyne C.M., Sanganalmath S.K., Langslet G., Baum S.J., Shah P.K., Koren A., Mandel J., Davidson M.H. (2018). Efficacy and Safety of Alirocumab in High-Risk Patients With Clinical Atherosclerotic Cardiovascular Disease and/or Heterozygous Familial Hypercholesterolemia (from 5 Placebo-Controlled ODYSSEY Trials). Am. J. Cardiol..

[11] Balakrishna A.M., Kaushik S., Palanivelu S.T., Monther N., Ponamgi S.P., Alla V.M., Patil S.M. (2025). Safety and Efficacy of Achieving Very Low LDL Cholesterol Concentrations with PCSK9 Inhibitors. J. Clin. Med..

[12] Krauss A. (2018). Why all randomised controlled trials produce biased results. Ann. Med..

[13] Shi X.Z., Qiao Y., Yang Y.L., Wang N.N., Zhang Y., Shi S.X., Shen G.B., Jia X.C. (2024). Mining of adverse event signals associated with inclisiran: A post-marketing analysis based on FAERS. Expert Opin. Drug Saf..

[14] Chen Z.H., Wang X., Teng Z.W., Huang J., Mo J.Z., Qu C.R., Wu Y.H., Liu Z.X., Liu F.K., Xia K. (2024). A comprehensive assessment of the association between common drugs and psychiatric disorders using Mendelian randomization and real-world pharmacovigilance database. Ebiomedicine.

[15] Nie H., Zhao W.P., Wang Q.Q., Zhou W.M. (2025). Lipid-lowering and antihypertensive drugs on aortic disease risk: Insights from Mendelian randomization analysis and real-world pharmacovigilance data. Eur. Heart J.-Cardiovasc. Pharmacother..

[16] van Puijenbroek E.P., Bate A., Leufkens H.G.M., Lindquist M., Orre R., Egberts A.C.G. (2002). A comparison of measures of disproportionality for signal detection in spontaneous reporting systems for adverse drug reactions. Pharmacoepidemiol. Drug Saf..

[17] Evans S.J.W., Waller P.C., Davis S. (2001). Use of proportional reporting ratios (PRRs) for signal generation from spontaneous adverse drug reaction reports. Pharmacoepidemiol. Drug Saf..

[18] Sakaeda T., Tamon A., Kadoyama K., Okuno Y. (2013). Data Mining of the Public Version of the FDA Adverse Event Reporting System. Int. J. Med. Sci..

[19] Graham S.E., Clarke S.L., Wu K.H.H., Kanoni S., Zajac G.J.M., Ramdas S., Surakka I., Ntalla I., Vedantam S., Winkler T.W. (2021). The power of genetic diversity in genome-wide association studies of lipids. Nature.

[20] Nikpay M., Goel A., Won H.H., Hall L.M., Willenborg C., Kanoni S., Saleheen D., Kyriakou T., Nelson C.P., Hopewell J.C. (2015). A comprehensive 1000 Genomes-based genome-wide association meta-analysis of coronary artery disease. Nat. Genet..

[21] Carter A.R., Sanderson E., Hammerton G., Richmond R.C., Smith G.D., Heron J., Taylor A.E., Davies N.M., Howe L.D. (2021). Mendelian randomisation for mediation analysis: Current methods and challenges for implementation. Eur. J. Epidemiol..

[22] Lee S., Lee W. (2025). A Review of Mendelian Randomization: Assumptions, Methods, and Application to Obesity-Related Diseases. J. Obes. Metab. Syndr..

[23] Nguyen K., Mitchell B.D. (2024). A Guide to Understanding Mendelian Randomization Studies. Arthritis Care Res..

[24] Deng Z.F., Liu J., Gong H.J., Cai X.A., Xiao H., Gao W.Q. (2024). Psychiatric disorders associated with PCSK9 inhibitors: A real-world, pharmacovigilance study. CNS Neurosci. Ther..

[25] Feng Z., Li X.Y., Tong W.K., He Q.F., Zhu X., Xiang X.Q., Tang Z.J. (2022). Real-world safety of PCSK9 inhibitors: A pharmacovigilance study based on spontaneous reports in FAERS. Front. Pharmacol..

[26] Ji C.M., Bai J.M., Zhou J.C., Zou Y., Yu M.M. (2022). Adverse event profiles of PCSK9 inhibitors alirocumab and evolocumab: Data mining of the FDA adverse event reporting system. Br. J. Clin. Pharmacol..

[27] Schmidt A.F., Finan C., Gordillo-Marañón M., Asselbergs F.W., Freitag D.F., Patel R.S., Tyl B., Chopade S., Faraway R., Zwierzyna M. (2020). Genetic drug target validation using Mendelian randomisation. Nat. Commun..

[28] Bell A.S., Rosoff D.B., Mavromatis L.A., Jung J., Wagner J., Lohoff F.W. (2022). Comparing the Relationships of Genetically Proxied PCSK9 Inhibition With Mood Disorders, Cognition, and Dementia Between Men and Women: A Drug-Target Mendelian Randomization Study. J. Am. Heart Assoc..

[29] Rosoff D.B., Bell A.S., Jung J., Wagner J., Mavromatis L.A., Lohoff F.W. (2022). Mendelian Randomization Study of PCSK9 and HMG-CoA Reductase Inhibition and Cognitive Function. J. Am. Coll. Cardiol..

[30] Seijas-Amigo J., Mauriz-Montero M.J., Suarez-Artime P., Gayoso-Rey M., Estany-Gestal A., Casas-Martínez A., González-Freire L., Rodriguez-Vazquez A., Pérez-Rodriguez N., Villaverde-Piñeiro L. (2023). Cognitive Function with PCSK9 Inhibitors: A 24-Month Follow-Up Observational Prospective Study in the Real World-MEMOGAL Study. Am. J. Cardiovasc. Drug.

[31] Gronich N. (2024). Central Nervous System Medications: Pharmacokinetic and Pharmacodynamic Considerations for Older Adults. Drugs Aging.

[32] Sportiello L., Capuano A. (2024). Sex and gender differences and pharmacovigilance: A knot still to be untied. Front. Pharmacol..

[33] Mosteoru S., Gaita D., Banach M. (2020). An update on PCSK9 inhibitors- pharmacokinetics, drug interactions, and toxicity. Expert Opin. Drug Met..

[34] Zeng W., Deng H., Luo Y., Zhong S., Huang M., Tomlinson B. (2024). Advances in statin adverse reactions and the potential mechanisms: A review. J. Adv. Res..

[35] Cesaro A., Bianconi V., Gragnano F., Moscarella E., Fimiani F., Monda E., Scudiero O., Limongelli G., Pirro M., Calabrò P. (2020). Beyond cholesterol metabolism: The pleiotropic effects of proprotein convertase subtilisin/kexin type 9 (PCSK9). Genetics, mutations, expression, and perspective for long-term inhibition. Biofactors.

[36] Kazibwe R., Rikhi R., Mirzai S., Ashburn N.P., Schaich C.L., Shapiro M. (2024). Do Statins Affect Cognitive Health? A Narrative Review and Critical Analysis of the Evidence. Curr. Atheroscler. Rep..

