# Supplementary material for: Do PCSK9 Inhibitors Impair Memory? A Dual Approach Combining Real-World Data and Genetic Evidence
Source: Pharmacy (Basel). 2025 Sep 3;13(5):125. doi: 10.3390/pharmacy13050125 (PMC12452292; doi:10.3390/pharmacy13050125)
Supplement: Supplementary file 1 [file pharmacy-13-00125-s001.zip › pharmacy-3818744-supplementary.pdf]

Supplementary Materials

Do PCSK9 Inhibitors Impair Memory? A Dual Approach Combining Real-World Data and Genetic Evidence

Xuezhong Shi [1], Shijia Wang [1], Yongli Yang [1], Xudong Xia [1,2], Jingwen Fan [1], Jingjing Wang [1], Nana Wang [1] and Xiaocan Jia [1,*]

[1] Department of Epidemiology and Biostatistics, College of Public Health, Zhengzhou University,
Zhengzhou 450001, China; xzshi@zzu.edu.cn (X.S.); sjwang2000@gs.zzu.edu.cn (S.W.); ylyang377@zzu.edu.cn (Y.Y.); hmpaxxd@126.com (X.X.); fjw@gs.zzu.edu.cn (J.F.); wjj95524@163.com (J.W.); wnn0924@126.com (N.W.)
[2] Center for Drug Evaluation of Henan, Zhengzhou 450000, China
* Correspondence: jxc@zzu.edu.cn

Table S1. 2×2 table of disproportionality analysis.

	Target AEs	Non-target AEs	Total
**PCSK9 inhibitors**	a	b	a+b
**Other drugs**	c	d	c+d
**Total**	a+c	b+d	a+b+c+d

Abbreviations: AEs: adverse events; a: the number of reports that include both the targeted drug and the associated AEs; b: the number of reports concerning other AEs related to the targeted drug; c: the number of reports involving other drugs associated with the targeted AEs; d: the number of reports that encompass both other drugs and unrelated AEs.

Table S2. The overview of the GWAS data.

Trait	Sample size (case/control)	Consortium or cohort study	Year of publication	Author	PubMed Identifier
**PCSK9 inhibitors**	146,492	GLGC	2021	Sarah E Graham	34887591
**CHD**	60,801/123,504	CARDIoGRAMplus C4D	2015	Majid Nikpay	26343387
**Memory loss**	3,377/472,508	FinnGen R12	2024	NA	NA
**Memory impairment**	5,175/310,493	Meta	2024	Anurag Verma	39024449
**Amnesia**	250/456,098	UK Biobank	2021	Longda Jiang	34737426

Abbreviations: CHD, coronary heart disease; GLGC, Global Lipids Genetics Consortium; CARDIoGRAM, Coronary ARtery DIsease Genome-wide Replication and Meta-analysis plus The Coronary Artery Disease (C4D) Genetics Consortium.

Table S3. The signal strength of memory loss for each PCSK9 inhibitor in the full dataset.

PCSK9 inhibitors	AEs	N	ROR (95%CI)	MHRA		EBGM05	IC025
				PRR	χ^2		
**Alirocumab**	Memory loss	51	1.37 (0.76-2.47)	1.37	1.09	0.83	-1.21
	Memory impairment	40	1.73 (1.27-2.36)	1.73	12.33	1.33	-0.88
	Amnesia	11	0.29 (0.22-0.38)	0.29	91.68	0.26	-3.29
**Evolocumab**	Memory loss	261	0.63 (0.55-0.71)	0.63	58.23	0.57	-2.34
	Memory impairment	204	0.67 (0.58-0.76)	0.67	33.98	0.60	-2.25
	Amnesia	55	0.52 (0.40-0.67)	0.52	24.85	0.42	-2.61
**Inclisiran**	Memory loss	77	1.86 (1.48-2.32)	1.85	30.20	1.53	-0.78
	Memory impairment	63	2.07 (1.61-2.65)	2.06	34.51	1.68	-0.62
	Amnesia	13	1.22 (0.71-2.11)	1.22	0.53	0.78	-1.37

Abbreviations: AEs, adverse events; CI, confidence interval; ROR, reporting odds ratio; MHRA, Medicines and Healthcare Products Regulatory Agency; PRR, proportional reporting ratio; EBGM05, lower limit of the 95% CI of the empirical Bayesian geometric mean; IC025, lower limit of the 95% CI of the information component.

Table S4. The signal strength of memory loss for each PCSK9 inhibitor in the lipid-lowering drugs dataset.

PCSK9 inhibitors	AEs	N	ROR (95%CI)	MHRA		EBGM05	IC025
				PRR	χ^2		
**Alirocumab**	Memory loss	51	1.32 (1.00-1.74)	1.31	3.78	1.04	-1.28
	Memory impairment	40	1.56 (1.14-2.13)	1.56	7.84	1.19	-1.04
	Amnesia	11	0.86 (0.47-1.56)	0.86	0.25	0.52	-1.89
**Evolocumab**	Memory loss	261	0.46 (0.41-0.52)	0.46	149.31	0.46	-2.64
	Memory impairment	204	0.55 (0.48-0.64)	0.55	60.08	0.53	-2.41
	Amnesia	55	0.29 (0.22-0.38)	0.29	91.68	0.26	-3.29
**Inclisiran**	Memory loss	77	1.51 (1.20-1.90)	1.51	12.91	1.24	-1.09
	Memory impairment	63	1.87 (1.46-2.41)	1.87	24.79	1.49	-0.78
	Amnesia	13	0.77 (0.44-1.32)	0.77	0.92	0.49	-2.05

Abbreviations: AEs, adverse events; CI, confidence interval; ROR, reporting odds ratio; MHRA, Medicines and Healthcare Products Regulatory Agency; PRR, proportional reporting ratio; EBGM05, lower limit of the 95% CI of the empirical Bayesian geometric mean; IC025, lower limit of the 95% CI of the information component.

Table S5. The stratification analysis of PCSK9 inhibitors in the full dataset.

Strata		AEs	N	ROR (95%CI)	MHRA		EBGM05	IC025
					PRR	χ^2		
**Age**	18 – 64	Memory loss	51	0.53(0.40-0.70)	0.53	21.38	0.42	-2.58
	years	Memory impairment	34	0.49(0.35-0.69)	0.49	17.70	0.37	-2.68
		Amnesia	16	0.61(0.38-1.00)	0.61	3.88	0.41	-2.37
	≥65	Memory loss	198	0.71(0.62-0.82)	0.71	22.42	0.64	-2.14
	years	Memory impairment	160	0.76(0.65-0.89)	0.76	11.68	0.67	-2.05
		Amnesia	38	0.59(0.43-0.81)	0.59	10.78	0.45	-2.42
**Gender**	Male	Memory loss	105	0.65(0.54-0.79)	0.65	19.19	0.56	-2.27
		Memory impairment	86	0.75(0.60-0.92)	0.75	7.32	0.63	-2.08
		Amnesia	18	0.42(0.26-0.66)	0.42	14.62	0.28	-2.92
	Female	Memory loss	266	0.84(0.74-0.94)	0.84	8.50	0.76	-1.92
		Memory impairment	211	0.88(0.77-1.01)	0.88	3.15	0.79	-1.84
		Amnesia	53	0.69(0.52-0.90)	0.69	7.44	0.55	-2.20

Abbreviations: AEs, adverse events; CI, confidence interval; ROR, reporting odds ratio; MHRA, Medicines and Healthcare Products Regulatory Agency; PRR, proportional reporting ratio; EBGM05, lower limit of the 95% CI of the empirical Bayesian geometric mean; IC025, lower limit of the 95% CI of the information component.

Table S6. The stratification analysis of PCSK9 inhibitors in the lipid-lowering drugs dataset.

Strata		AEs	N	ROR (95%CI)	MHRA		EBGM05	IC025
					PRR	χ^2		
**Age**	18 – 64	Memory loss	51	0.33(0.25-0.43)	0.33	66.95	0.29	-3.13
	years	Memory impairment	34	0.35(0.25-0.50)	0.35	38.42	0.29	-3.03
		Amnesia	16	0.28(0.17-0.46)	0.28	28.93	0.20	-3.36
	≥65	Memory loss	198	0.69(0.59-0.80)	0.69	23.83	0.65	-2.11
	years	Memory impairment	160	0.86(0.72-1.02)	0.86	3.09	0.77	-1.85
		Amnesia	38	0.39(0.28-0.54)	0.39	33.23	0.34	-2.83
**Gender**	Male	Memory loss	105	0.51(0.41-0.62)	0.51	45.16	0.47	-2.51
		Memory impairment	86	0.65(0.52-0.81)	0.65	14.43	0.57	-2.20
		Amnesia	18	0.25(0.16-0.41)	0.25	37.88	0.20	-3.46
	Female	Memory loss	266	0.63(0.55-0.72)	0.63	48.45	0.62	-2.21
		Memory impairment	211	0.76(0.65-0.88)	0.76	13.43	0.71	-1.99
		Amnesia	53	0.38(0.29-0.50)	0.38	48.78	0.35	-2.86

Abbreviations: AEs, adverse events; CI, confidence interval; ROR, reporting odds ratio; MHRA, Medicines and Healthcare Products Regulatory Agency; PRR, proportional reporting ratio; EBGM05, lower limit of the 95% CI of the empirical Bayesian geometric mean; IC025, lower limit of the 95% CI of the information component.
